# Analytical Study of Fuel Switching from Heavy Fuel Oil to Natural Gas in clay brick factories at Arab Abu Saed, Greater Cairo

**DOI:** 10.1038/s41598-019-46587-w

**Published:** 2019-07-12

**Authors:** Mamdouh Higazy, Khaled S. M. Essa, Fawzia Mubarak, El-Sayed M. El-Sayed, Abdelsattar M. Sallam, Mona S. Talaat

**Affiliations:** 1 Biophysics Group, Department of Physics, Faculty of Science, Ain Shams University, Cairo, Egypt; 2 Mathematics and theoretical physics Department, Nuclear Research Center, Egyptian Atomic Energy Authority, Cairo, Egypt; 3 Radiation Protection Department, Nuclear Research Center, Egyptian Atomic Energy Authority, Cairo, Egypt

**Keywords:** Physical sciences, Physics, Applied physics

## Abstract

Arab Abu Saed area in Giza governorate, south to Cairo contains more than 228 clay brick kilns represent the largest cluster of brickworks in Egypt. Burning of Heavy Fuel Oil (HFO) in such kilns is the main source of air pollution in the surrounding locations. In this study, investigation of switching the fuel used in brick kilns from (HFO) to Natural Gas (NG) is carried out and the pollution loads are assessed in both cases. In addition, two Gaussian dispersion plume models are employed to estimate the concentration of primary pollutants; PM_10_, SO_2_, and NO_2_ at seven locations in the vicinity of Arab Abu Saed to determine the most adversely affected locations. Statistical analysis is applied to evaluate the correlation and conformity of the results of both models. Results show that using of NG leads to a significant reduction of pollution loads of PM_10_, SO_2_ and NO_2_ reaches 96%, 72%, and 24% respectively. In addition, the reduction of naturally occurring radionuclides in air is analyzed. Activity concentrations of Ra-226, Th-232 and K-40 in Bq/l for HFO were measured using HPGe detector for six HFO samples. Exposure due to air submersion of naturally occurring radionuclides in the study area leads to annual equivalent dose ranged between 2.16 mSv/y (received by Uterus) and 14 mSv/y (received by skin), and average effective dose 2.65 mSv/y which represent valuable exposure.

## Introduction

Greater Cairo suffers from a serious air quality problem resulting from the release of different air pollutants with high emission loads. The largest contributors to the air pollution in Cairo are the industrial activities, energy production, transportation, and waste burning^1,2^. Most of air pollutants have significant health impact on the human being, especially PM_10_, SO_2_, and NO_2_. In 2013, the specialized cancer agency of the World Health Organization (WHO), the International Agency for Research on Cancer (IARC), announced that outdoor air pollution has been classified as carcinogenic to humans^3^. About 7 million deaths in the world per year were reported by the WHO due to air pollution^4^. However, the Egypt State of the Environment Report 2015 showed that the annual mean concentrations of PM_10_, SO_2_, and NO_2_ (148, 23, and 51 μg/m^3^ respectively) were complying with the Egyptian permissible limits; these concentrations were exceeding the WHO air quality standards (20, 20, 40 μg/m^3^)^1–5^. In June and October 2010, The Egyptian 24 h PM_10_ standard of 70 μg/m^3^ was exceeded during 91 and 96% of the sampling periods, respectively, in all studied sites in Greater Cairo. Resuspended geological dust, mobile source emissions, and open (vegetative/trash) burning were the major contributors to PM_10_ levels^6^. In addition, the mean annual concentrations of PM_10_ in Greater Cairo from 16 April 2014 to 29 January 2015 reveals that about 70% of the region is considered as unhealthy for sensitive groups (200–250 µg/m^3^ year)^2–7^.

Arab Abu Saed area is located approximately 40 km south to Cairo. This area contains more than 228 clay brick kilns which represent the largest cluster of brickworks in Egypt. All the brick factories are identically operated using the same old traditional Open-Hoffman kilns where fuel is combusted^8^. These kilns are designed to allow the movement of the combustion air and the combustion exhaust by the natural drift caused by tall stacks (60–80 m)^9^. The Heavy Fuel Oil (HFO) and the Natural Gas (NG) are the only available types of fuels to be used in these kilns.

The aim of this paper is studying the impact on the air quality due to conversion of the fuel used from HFO to NG in 176 brick kilns. The assessment is focusing on three primary air pollutants; PM_10_, SO_2_, and NO_2_ in seven different around locations (five towns and two monitoring stations) namely; Ikhsas, Barnasht, Tabbin, Minya, and Qibliya Towns, and Tabbin South Ambient Air Quality (AAQ) monitoring station, and Tabbin AAQ monitoring station. PM_10_, SO_2_, and NO_2_ are selected due to their massive negative contribution in the Greater Cairo air quality. To overcome the dilemma of carrying out detailed stack measurements for 176 stacks, specific emission factors are developed for this type of kilns based on the type of fuel. These emission factors were used to estimate the emission rates (g/s) of the target primary pollutants. Then, two Gaussian plume models, Classical GPM (CGPM) and American Environmental Regulatory Model (AERMOD), are typically used to study and understand the variation in the pollutants’ concentration and pollution loads on the surroundings and the vulnerable communities due to fuel switching. GPM requires knowledge of source characteristics, terrain and meteorology^10^. It has different levels and versions starting from the classical equation to the recent advanced models. It is often advised to apply such a classical model prior to using the more advanced models^11,12^. AERMOD is used for simulation of the dispersion of air pollutants. Because of its ability to run with minimally observed meteorological parameters, it is considered an ideal model for many African countries suffering from difficulty in collection of metrological data^13^. The CGPM was used to preliminary assess the pollutant concentrations at the target locations, while AERMOD was applied to verify the results through considering extra atmospheric parameters and the surrounding terrain. Also, radiation measurements were carried out for six HFO samples to investigate the dispersion of occurring radionuclides in air resulting from burning the HFO and calculate the annual average effective dose to workers or public to assess the radiological hazard.

## Results and Discussions

Based on the detailed measurements and the surveyed data, the results of the emission factors’ calculations are; in case of using HFO 11380, 3115, and 979 g/1000 RB for PM_10_, SO_2_, and NO_2_ respectively, while for NG the emission factors are 456, 864, 744 g/1000 RB for PM_10_, SO_2_, and NO_2_ respectively. Comparing with a similar study in Greater Dhaka region, Bangladesh^14^, in which coal is the dominant fuel, results show that the coal emission factors are 9700, 4600, 4700 g/1000 RB for PM_10_, SO_2_, and NO_2_ respectively. Consequently, it is concluded that burning HFO causes the highest levels of PM_10_, while the highest levels of SO_2_ and NO_2_ are released due to using coal.

The fuel switching from HFO to NG in Arab Abu Saed brickworks cluster has resulted in a significant emission load reduction (Table 1) of primary pollutants PM_10_, SO_2_, and NO_2_ that reached 96%, 72%, and 24% respectively. This reduction has been resulted mainly due to the difference in the chemical composition between the HFO and NG and subsequently the combustion exhaust. NG is composed mainly of methane, where the main products of combustion are carbon dioxide and water vapor. HFO has more complex content with higher nitrogen, sulfur, and carbon ratio. Thus, combustion of HFO releases higher levels of harmful emissions, NO_2_, SO_x_ and ash particles. The combustion of NG, on the other hand, releases very small amounts of sulfur dioxide and nitrogen oxides, virtually no ash or particulate matter^15,16^. However, the emission loads resulting from using NG is considered very low compared to the HFO loads, using the biomass fuel in the brick kilns (44 kilns) at Bang Pu area in southern Thailand^17^ has resulted in much fewer emission loads 71 and 363 ton/year of PM_10_ and SO_2_ respectively.

**Table 1:** Emission loads of primary pollutants.

Fuel	No. of Kilns	Emission Loads (ton/year)		
		PM_10_	SO_2_	NO_2_
HFO	176	48,552	13,290	4,177
NG	176	1,945	3,686	3,174
Reduction (t/y)		46,607	9,604	1,003
Reduction (%)		96%	72%	24%

### Results of the dispersion models

The estimation of the highest concentration at 1-hr average of the target air pollutants was done for both scenarios of burning the HFO and NG in the brick kilns. The details of the two Gaussian dispersion models’ results are presented in Table (2), it shows that Minya town has the highest concentration of PM_10_, SO_2_, and NO_x_ in case of burning HFO, while the lowest concentration is estimated to be at Tabbin South monitoring station if NG is used. However, Minya town is the most positively affected site of the pollution reduction resulting from the fuel switching.

**Table 2:** CGPM and AERMOD estimation of pollutants’ concentrations at the target locations.

Location	Dispersion Model	Pollutants’ concentrations (μg/m^3^)/hour					
		PM_10_		SO_2_		NO_2_	
		HFO	NG	HFO	NG	HFO	NG
Ikhsas town	CGPM	1508.3	60.4	949.26	114.52	298.34	226.72
	AERMOD	1356.4	52.45	1309.2	99.404	358.4	229.18
Barnasht town	CGPM	1102.7	44.2	963.09	83.72	302.7	230.0
	AERMOD	1172.9	45.34	1131.9	85.937	309.8	198.13
Tabbin town	CGPM	1137.3	23.7	691.30	86.35	217.27	145.5
	AERMOD	847.87	32.77	818.91	62.12	223.96	143.22
Minya town	CGPM	1788.9	71.6	1127.9	135.8	354.4	269.4
	AERMOD	1744.3	67.43	1683.2	127.8	460.75	294.65
Qibiliya town	CGPM	935.40	37.5	890.38	71.02	279.8	212.6
	AERMOD	1567.7	60.6	1512.8	114.86	414.12	264.82
Tabbin South Monitoring Station	CGPM	529.21	21.2	1516.2	192.85	476.52	273.77
	AERMOD	1723.3	66.62	1663.0	126.26	455.2	291.11
Tabbin Ambient Monitoring Station	CGPM	1064.23	40.92	951.95	77.55	299.18	147.46
	AERMOD	858.08	33.172	828.04	62.87	226.6	144.95

The predicted 1-hr average concentrations in μg/m^3^ of PM_10_, SO_2_, and NO_x_ in case of burning HFO and NG show similarity of the distribution and the locations of the high and low concentrations of the primary pollutants in both scenarios was expected due to the flat terrain of Arab Abu Saed area and the anticipated low wind speed. Comparing the estimated emissions with other studies in the same domain but using different types of fuels (Skinder *et al*. (2014), Kanabkaew *et al*. (2015), and Hoang Anh Le *et al*. (2010))^17–19^, revealed that using biomass fuel is the cleanest way of clay brick production. NG is considered the best alternative in case of burning fossil fuel, while HFO and coal are the worst.

### Statistical analysis

**Figure 1:**
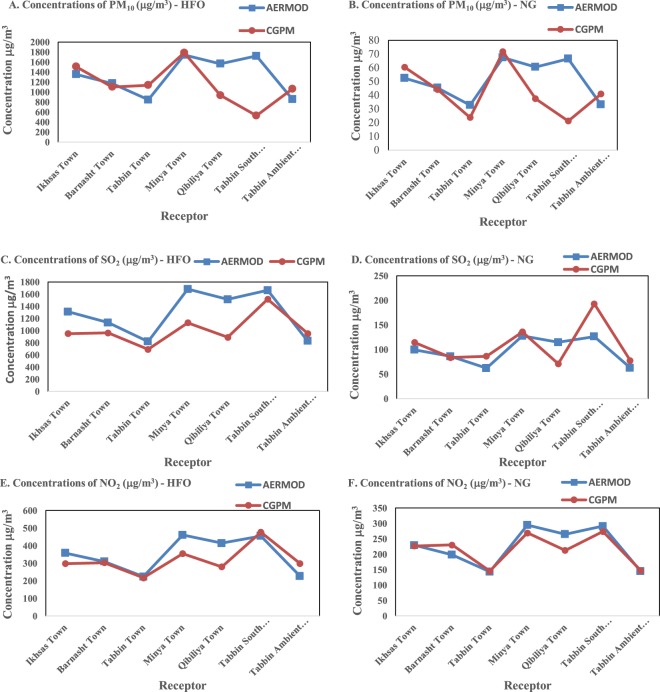
GCPM and AERMOD results of pollutants’ concentrations (the highest1-hr average concentration).

Based on the results given by CGPM and AERMOD, a comparison and statistical analysis were carried out to assess the correlation and conformity between both models. Figure 1 presents the comparison between GCPM and AERMOD results of the highest 1-hr average concentrations of the target pollutants. Table (3) confirmed the correlation and the agreement of both models’ results. Consequently, it is concluded that, in case of uncomplicated (flat) surrounding terrain and low wind speed condition, using Power law scheme to calculate the plume dispersion parameters in CGPM can give accurate results compared to the advanced Gaussian Plume models like AERMOD.

**Table 3:** Standard statistical performance of CGPM and AERMOD.

Pollutant	Type of Fuel	NMSE	FB	COR	FAC2
PM_10_	HFO	0.25	0.18	−0.48	0.92
	NG	0.18	0.18	0.30	0.87
SO_2_	HFO	0.10	0.23	0.7	0.82
	NG	0.18	0.11	0.30	0.94
NO_2_	HFO	0.07	0.13	0.66	0.92
	NG	0.02	0.03	0.89	0.98

### Radioactivity concentration and equivalent dose

The activity concentrations (Bq/l) of Ra-226, Th-232 and K-40 for six samples of HFO are presented in Table (4). It shows that activity concentrations ranged between 1.0 to 1.9, 1.0 to 1.7 and 1.0 to 2.5 Bq/l for Ra-226, Th-232 and K-40 respectively. Although activity concentration is considered low for all radionuclides per one liter, but the daily HFO consumption of each kiln (average 5000 liter per day) leads to dispersion of a considerable activity concentration of Ra-226, Th-232 and K-40 that reach 7000, 6500, and 8000 Bq/day respectively. The calculated annual occupational exposure of Arab Abu Saeed workers is presented in Table (5), in which the annual external dose due to average concentrations of all natural radionuclides ranged between 2.16 (received by Uterus) and 14 mSv/y (received by skin). The average effective dose due to exposure to all radionuclides is 2.65 mSv/y.

**Table 4:** Activity concentrations (Bq/l) of HFO samples.

Sample	Bq/l		
	Ra-226	Th-232	K-40
S1	1.0	1.0	2.5
S2	1.9	1.4	1.0
S3	1.7	1.2	1.0
S4	1.0	1.7	1.9
S5	1.5	1.2	1.8
S6	1.4	1.5	1.5
Average	1.4 ± 0.4	1.3 ± 0.2	1.6 ± 0.6
Range	1–1.9	1–1.7	1–2.5
Daily concentration (Bq/day)	7000	6500	8000

**Table 5:** Annual equivalent dose and effective dose due to average activity (mSv/y).

Organ	Ra-226	Th-232	K-40	Summation
R Marrow	3.62E-04	1.50E-03	2.63E + 00	2.63E + 00
Adrenals	1.72E-04	1.23E-03	2.22E + 00	2.22E + 00
B Surface	2.16E-03	7.06E-03	3.71E + 00	3.72E + 00
Brain	2.32E-04	1.64E-03	2.84E + 00	2.84E + 00
Breast	2.50E-03	3.69E-03	2.95E + 00	2.96E + 00
G Bladder	1.60E-04	1.20E-03	2.25E + 00	2.26E + 00
Esophagus	1.23E-04	1.07E-03	2.29E + 00	2.29E + 00
ST Wall	2.41E-04	1.45E-03	2.39E + 00	2.39E + 00
SI Wall	1.43E-04	1.13E-03	2.21E + 00	2.21E + 00
ULI Wall	1.65E-04	1.23E-03	2.26E + 00	2.26E + 00
LLI Wall	1.45E-04	1.15E-03	2.23E + 00	2.24E + 00
Heart	2.18E-04	1.38E-03	2.37E + 00	2.38E + 00
Kidneys	3.13E-04	1.55E-03	2.39E + 00	2.40E + 00
Liver	2.37E-04	1.48E-03	2.42E + 00	2.42E + 00
Lungs	2.91E-04	1.73E-03	2.64E + 00	2.64E + 00
Ovaries	1.27E-04	1.06E-03	2.27E + 00	2.27E + 00
Pancreas	1.28E-04	1.09E-03	2.18E + 00	2.19E + 00
Skin	8.50E-03	9.34E-03	1.40E + 01	1.40E + 01
Spleen	2.19E-04	1.46E-03	2.42E + 00	2.42E + 00
Testes	1.28E-03	2.53E-03	2.61E + 00	2.61E + 00
Thymus	3.45E-04	1.68E-03	2.54E + 00	2.54E + 00
Thyroid	7.95E-04	2.14E-03	2.70E + 00	2.70E + 00
U Bladder	2.31E-04	1.37E-03	2.25E + 00	2.26E + 00
Uterus	1.34E-04	1.09E-03	2.16E + 00	2.16E + 00
Muscle	1.08E-03	2.25E-03	2.58E + 00	2.58E + 00
h_rem_	9.97E-04	2.17E-03	2.58E + 00	2.58E + 00
E	7.31E-04	1.96E-03	2.65E + 00	2.65E + 00
Min	**1.23E-04**	**1.06E-03**	**2.16E + 00**	**2.16E + 00**
Max	**8.50E-03**	**9.34E-03**	**1.40E + 01**	**1.40E + 01**

## Materials and Methods

To assess the anticipated improvement resulting from the fuel-switching, CGPM and AERMOD are used and two scenarios are set-up. The first scenario is considering the usage of the HFO in the 176 brick kilns, and measures the pollutants’ concentrations at the target locations. The second one calculates the pollutants’ concentrations at the same locations in case of burning NG in the brick kilns. The operation of AERMOD and CGPM require obtaining specific data concerning pollutants’ emission rates, flue gas velocity, the downwind and lateral distances between the source and receptors, terrain and atmospheric data. The methodology for getting the required data relies on conducting field surveys, carrying out detailed monitoring and measurements, and the relevant previous work in the area which includes; CIDA-pilot project^9^.

### Emission factors and Emission rates

Owing to the identical similarity of the production process of Arab Abu Saed brickworks and the difficulties of conducting real measurements for that high number of stacks, it was found that developing emission factors based on the size of production and the type of fuel would be very efficient to elicit emission rates from the stacks of all kilns. Accordingly, to calculate the emission factors, several parameters have to be determined including concentrations of pollutants, the emissions flow rates, the specific consumption of fuel, and the production capacity. Through the conducted field surveys, the amount of fuel used and the production capacity per each kiln were collected. In addition, detailed monitoring and measurements were done for a sample of two kilns per each type of fuel to determine the concentrations of air pollutants and the flow rates (summary of these measurements are presented in Table (6).

**Table 6:** cDetailed measurement of stack emissions for two factories burning HFO and other burning NG.

Emission Parameter	Heavy Fuel Oil (HFO)			Natural Gas (NG)		
	Kiln 1	Kiln 2	Average	Kiln 3	Kiln 4	Average
Production (tone/day)	141.8	138.5	140.15	166.8	150.1	158.45
Heat released (MMBTU/hr)	8.73	7.50	8.11	11.01	11.24	11.12
Stack gas flow rate (m^3^/hr)	22796	13839	18317.5	24131	32937	28534
Stack gas temperature (C)	87	113	100	84.1	80	82.55
Stack gas moisture (%)	7.44	8.96	8.2	7.78	7.23	7.5
Stack gas oxygen (% dry)	18	17.7	17.85	18.9	18.8	18.85
PM conc. (mg/m^3^)	1679	1714	1696.5	74.1	61.5	67.8
PM_10_ % of PM	95	97	96	38.5	48.6	43.55
SO_2_ conc. (mg/m^3^)	250	618	434	83	82	82.5
NO_2_ conc. (mg/m^3^)	69	209	139	75	66.8	70.9
Average production (Red Brick) RB/day	64,571	72,232	68,401	69,200	58,532	63,866
Average Consumption/HFO (kg/day) – NG (m^3^/day)	5,749	6,303	6,026	6,426	5,697	6,035
Specific Fuel consumption/HFO (kg/1000 RB) – NG (m^3^/1000 RB)	89.03	87.26	88.14	101	115	108

The mass emissions per unit time (g/s), unit production (g/ton of Red Brick (RB)) and unit energy used (lb/MBTU) are the basis of calculating the emission factors in terms of (g/ton RB), however this unit has been converted into another easier unit i.e. (g/1000 RB). The objective is to consider the variation of brick size by using the average density of the produced red bricks in both sets of measurements. The average density of bricks produced by burning HFO is 2.3 kg/RB, while the average density is 2.4 kg/RB if NG is burnt. The resulting Emission factors in case of using HFO are 11380, 3115, and 979 g/1000 RB for PM_10_, SO_2_, and NO_2_ respectively, while for NG the Emission factors are 456, 864, 744 g/1000 RB for PM_10_, SO_2_, and NO_2_ respectively. Thus, according to the fuel used, the emission load and the subsequent emission rate for each pollutant per each kiln stack are calculated using the following general formula:

1 $$Pollution\,load\,(\frac{g}{y})=Production\,size\,(\frac{No.\,of\,RB}{1000})\times Emissionfactor\,(EF)\,(\frac{g}{1000RB})$$

and the pollution load of e.g. PM_10_ for the cluster is estimated as follows:

2 $${{\rm{PM}}}_{10}=\sum _{i=1}^{n}{({\rm{Production}})}_{{\rm{i}}}\times {{\rm{EF}}}_{P{M}_{10}}$$

3 $$Emission\,rate\,(\frac{g}{s})=\frac{Annual\,Emission\,Load(\frac{g}{y})}{350\frac{day}{y}\times 24\frac{hr}{day}\times \frac{3600\,sec}{hr}}$$

The velocity of flue gases (m/s) is essential input for both models. The calculation depends on the measured volume flow rate (m^3^/s), fuel consumption rate, and the cross section area of the stack (m^2^). The general formula for calculating the flue gas velocity (v m/s) is;

4 $$v(\frac{m}{s})=\frac{Exhaust\,flow\,rate(\frac{{m}^{3}}{s})}{Stack\,cross\,section\,area\,({m}^{2})}$$

### Classical gaussian plume model (CGPM)

Conventional Gaussian plume model is commonly used for the air quality assessment and pollution analysis activities^20,21^. The Gaussian models are based on the solution of an advection diffusion equation derived by assuming that the wind speed and eddy diffusivity do not have spatial and temporal variations^22,23^. The following formula represents the concentration of a pollutant releasing from a continuous source.

5 $$C(x,y,z,H)=\frac{Q}{2\pi {\sigma }_{y}{\sigma }_{z}U}{e}^{(-\frac{{y}^{2}}{2{\sigma }_{y}^{2}})}\{{e}^{(-\frac{{(z-H)}^{2}}{2{\sigma }_{z}^{2}})}+{e}^{(-\frac{{(z+H)}^{2}}{2{\sigma }_{z}^{2}})}\}$$

where, C is the concentration of the pollutant at a point with coordinates x, y, z (g/m^3^),

x, y & z are the downwind, lateral & vertical distances from the source (m) respectively,

Q is the emission rate (g/s),

U is the downwind velocity (m/s),

H is the effective source height above the ground (m),

σ_y_ & σ_z_ are the plume dispersion parameters in the lateral direction and vertical directions respectively.

The CGPM requires the calculation of the downwind velocity at the exit height of the flue gases. Since the average stack height of the cluster is 75 meter, the downwind velocity U_75_ is obtained by;

6 $${{\rm{U}}}_{75}={{\rm{U}}}_{10}{(\frac{{h}_{s}}{10})}^{p}$$

where, U_75_ is the wind speed at 75 m height.

U_10_ is the wind speed at 10 m height.

h_s_ is the physical stack height (75 m).

p is a stability class parameter.

H is the effective height above the ground (m) and is generally presented in the form;

7 $$\begin{array}{cc}H={h}_{s}+{\rm{\Delta }}h, & {\rm{\Delta }}h=3(\frac{w}{{U}_{75}})D\end{array}$$

where, h_s_ is the physical stack height,

Δh is the plume rise (m),

w is the exits velocity,

D is the internal stack diameter.

σ_y_ and σ_z_ can be calculated using different schemes. However, according to^22^, the power law scheme was found to give accurate results in case of U_75_ is greater than 2 m/s.

8 $${\sigma }_{y}=a{x}^{b}$$

9 $${\sigma }_{z}=c{x}^{d}$$

where, a, b, c, and d are Brookhaven National Laboratory Parameters which are determined based on the stability classes. Table 7 presents the assumptions used in the Classical Gaussian plume model calculations of the pollutants’ concentrations at the target locations.

**Table 7:** CGPM Assumptions used for the calculations of the pollutants’ concentrations.

Input	Value	unit
Physical average Stack Height (h_s_)	75	m
Stack Inside Diameter (D)	1.43	m
Stack gas Exit Velocity (w)	4.7	m/s
Stability Class	neutral	
Gas Exit temperature (T)	368	K
Wind speed at 10 m height (U_10_)	4.5	m/s
Wind speed at 75 m height (U_75_)	6.09	m/s
Stability classes (P)	0.15	
The plume rise Δh	2.71	m
The effective source height (H)	77.71	m

### AERMOD

AERMOD is an advanced new generation model developed by US-EPA mainly for regulatory proposes, and it is commonly used to study the impact of new or existing industrial facilities as well. It can be applied with different types of emission sources (i.e. point, area, or volume sources) and different plume types^24,25^. It is recommended to be used in Arab Abu Saed case due to its ability to parameterize turbulence and handling simple or complex terrain. The Breeze AERMOD software version (7.0.58) is employed to simulate the PM_10_, SO_2_, and NO_2_ concentrations at the target locations. It uses Geographical Information System (GIS) based approach to define the model objects, add the terrain relief, and present the results. Consequently, in order to set up the model and run the AERMOD, there are several options required to be filled as discussed below.

### Meteorology

**Figure 2:**
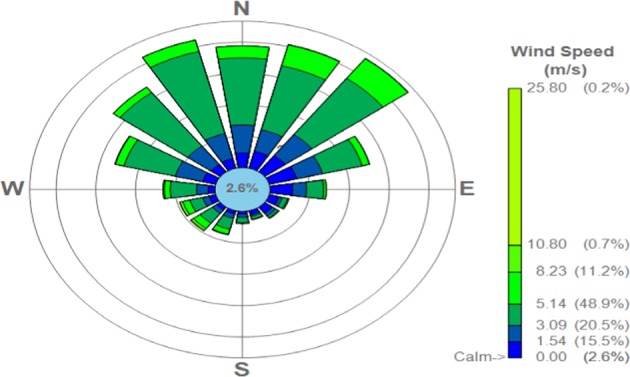
Wind rose of Cairo region.

AERMOD requires comprehensive meteorological information covering two types of data, one is a surface profile (typically available from the ground weather stations) and the other is the upper surface data (for parameters such as Monin-Obukhov length, albedo, bowen ratio etc. based on radiosonde observations). The Wind rose of the Cairo region based on the annual meteorological data (Fig. 2) was developed in the AERMOD required format. The height of the anemometer instrument is assumed 10 m. The wind rose shows the wind speeds range between 3 to 8 m/s in most periods of the year. Approximately half a year the wind speed reaches 5.14 m/s. The highest frequency wind direction was the northeast.

### Projection

For setting up the geographical location (latitude and longitude) of the sources and receptors in this model, the Universal Transverse Mercator (UTM) in the World Geodetic system (WGS1984) was used with zone 36 in the northern hemisphere (for Cairo, Egypt). Most of the terrain in and around the cluster is flat, although the terrain on the north and east is moderately complex (hilly) and of heights up to 145 m on the east. Since most of the terrain in the immediate vicinity are less than 10 times the average stack height, terrain do not have a considerable effect on the ambient path of the pollutants released from the stacks of the brick kilns^23–26^.

The sources of pollutant emissions (176 kiln stacks) were defined on the airshed map using their latitude and longitude. The worst case scenario was designed where pollutants were considered to be released in a constant rate throughout the year without any emission variations.

### Receptor options

Seven target receptors were selected and defined on the airshed map. The receptors were placed in the surrounding vulnerable communities and represented different categories such as urban town areas, farmlands, desert areas, and mixed land use areas, as well. The locations of these receptors are; Ikhsas town, Barnasht town, Tabbin town, Minya town, Qibliya town, Tabbin South AAQ station, and Tabbin AAQ station.

### Output options

Two scenarios were set-up to assess the impact of the fuel switching in Arab Abu Saed brickworks clusters; the first one considered the burning of the HFO, while the second scenario showed the result of the fuel switching to NG. In both scenarios, the 1-hr average concentrations of PM_10_, SO_2_, and NO_2_ were predicted in the target receptors. Based on the results of afore-elucidated calculation treatments and the survey outputs, Table (8) represents a sample of the input data used in both models, CGPM and AERMOD, for five brick kilns. These data are calculated for the entire cluster (i.e. 176 brick kilns) in order to obtain the substantial environmental impact of the fuel switching.

**Table 8:** Sample of input data used for the calculation of the annual average concentrations of the primary pollutants.

Kiln code	Longitude	Latitude	Stack height	Stack Diameter	Flue gas velocity	Pollutants Emission rates					
						PM_10_ (HFO)	SO_2_ (HFO)	NO_2_ (HFO)	PM_10_ (NG)	SO_2_ (NG)	NO_2_ (NG)
	GPS-N	GPS-E	m	m	m/s	g/s	g/s	g/s	g/s	g/s	g/s
k-1	29°45′38.23″N	31°19′42.87″E	90	1.1	6.49	9.88	2.70	0.85	0.396	0.75	0.646
k-20	29°45′51.96″N	31°20′30.35″E	85	1.3	8.13	11.20	3.06	0.96	0.448	0.85	0.732
k-87	29°44′29.48″N	31°20′40.39″E	70	1.6	4.60	8.56	2.34	0.74	0.343	0.65	0.560
k-116	29°45′13.67″N	31°21′11.44″E	55	1.5	4.36	7.24	1.98	0.62	0.290	0.55	0.474
k-176	29°43′59.42″N	31°21′16.01″E	85	1.6	3.83	9.22	2.52	0.79	0.369	0.7	0.603

### Statistical analysis

Different statistical parameters were used to analyze the correlation and agreement between both models. These parameters are Normalized Mean Square Error (NMSE), Fractional Bias (FB), Correlation Coefficient (COR), and Factor of two (FAC2)^27,28^.

10 $${\rm{Normalized}}\,{\rm{Mean}}\,{\rm{Square}}\,{\rm{Error}}\,({\rm{NMSE}})=\frac{\overline{{({{\rm{C}}}_{{\rm{p}}}-{{\rm{C}}}_{{\rm{o}}})}^{2}}}{\overline{({{\rm{C}}}_{{\rm{p}}}{{\rm{C}}}_{{\rm{o}}})}}$$

11 $${\rm{Fraction}}\,{\rm{Bias}}\,({\rm{FB}})=\frac{(\overline{{C}_{o}}-\overline{{C}_{p}})}{[0.5(\overline{{C}_{o}}+\overline{{C}_{p}})]}$$

12 $${\rm{Correlation}}\,{\rm{Coefficient}}\,({\rm{COR}})=\frac{1}{{N}_{m}}\sum _{i=1}^{{N}_{m}}\,({C}_{pi}-\overline{{C}_{p}})\times \frac{({C}_{oi}-\overline{{C}_{o}})}{({\sigma }_{p}{\sigma }_{o})}$$

13 $${\rm{Factor}}\,{\rm{of}}\,{\rm{Two}}\,({\rm{FAC}}2)=0.5\le \frac{{{\rm{C}}}_{{\rm{p}}}}{{{\rm{C}}}_{{\rm{o}}}}\le 2.0$$

In this case, the CGPM results are considered the predicted results (C_p_ = C_CGPM_/Q), while the observed results are the outputs of the AERMOD (C_o_ = C_AERMOD_/Q). σ_p_ and σ_o_ are the standard deviations of predicted $\overline{{C}_{p}}$ and observed $\overline{{C}_{o}}$ normalized crosswind integrated concentration respectively. Over bars indicate the average over all measurements. For perfect-correlated models, NMSE and FB should be equal or close to zero, while COR and FAC2 should be equal or close to one.

### Radiation measurement

The aim of the radiation measurement is to assess the radiological impact of burning the HFO in the brick kilns and determine the annual effective equivalent dose to the workers due to the exposure to the resulting radionuclides.

### The activity concentrations

Six HFO samples were collected randomly from different Brick kilns to measure and calculate the activity concentrations (Bq/l) of Ra-226, Th-232 and K-40. Each sample was packed in plastic cylindrical container; closed tightly and kept for about one month to attain secular equilibrium between Ra −226 and its progenies before exposing to gamma spectroscopy. The γ-spectroscopy used was P-type coaxial (HPGe) with 30% relative efficiency and 2.1 keV FWHM at 1.33 MeV (associated with electronic components), and connected to multi-channel analyzer (MCA). GENE 2000 software program was used to analyze the peak areas of gamma spectra^29^.

Activity concentration (A) in Bq/l of each radionuclide in the sample was calculated by using the count rates detected by the HPGe for 24 hours and subtracting the background counts under the selected photo peaks using the following equation;

14 $${\boldsymbol{A}}=\frac{{{\boldsymbol{N}}}_{{\boldsymbol{c}}}}{{\boldsymbol{\varepsilon }}\times {{\boldsymbol{I}}}_{{\boldsymbol{\gamma }}}\times (\frac{{\boldsymbol{m}}}{{\boldsymbol{\rho }}})}$$

where, N_c_ is the net count rate and equals to (Gross counts per second from the samples - background counts per second),

ε is the efficiency of the detector for the specific energy,

I_γ_ is the intensity of the gamma ray,

m is sample mass in gm,

ρ is the density of the HFO sample in gm/l.

#### External equivalent and effective organ doses

By using the activity concentration of different radionuclides, the external dose to an organ/tissue H_t_ (mSv/y) can be determined using the specific external dose coefficients for each organ/tissue; h_t_ (Sv.m^3^/sec.Bq), that were calculated by Eckerman and Ryman^30^ through DFEXT code; Federal Guidance Report No. 12.

15 $${H}_{T}=A\times T\times 3600(sec/hr)\times {h}_{t}\times 1000$$

where, T is the exposure time (5 day/week *50 week/year for workers),

For the remainder tissues, the committed equivalent dose H_rem_ can be calculated using the external dose coefficient of remainder tissues h_rem_ which is given 0.2 Sv.m^3^/sec.Bq. The effective dose (E) is the sum of the weighted equivalent doses in all tissues and organs of the body, and can be calculated as:

16 $$E=\sum _{T}{H}_{T}{W}_{T}$$

where, W_T_ is the weighting factor of tissue T used to represent the relative contribution of the equivalent dose in a tissue or organ to the total radiation hazard resulting to the body. W_T_ is found in ICRP-60^31^.

## Conclusion

The main source of air pollution in Arab Abu Saed Brickworks cluster is burning the fuel in the brick kilns. Switching the used fuel from HFO to NG has resulted in a significant load reduction of air pollutants. By calculating the emission rates of PM_10_, SO_2_, and NO_2_, it is found that fuel switching has led to substantial reductions in sulphur dioxide and particulate (respirable) concentrations in the ambient, while Nitrogen dioxide concentrations have reduced but to a slightly lesser extent. The load reduction of PM_10_, SO_2_, and NO_2_ has reached to 96%, 72%, and 24% respectively and this will considerably reduce exposure levels in the near vicinity and bring in health benefits to the nearby population. We can conclude that the reduction in exposure level and the health benefits will occur due to fuels change overall other industries sources and mobile sources rather than brick kilns only Two Gaussian plume models, CGPM and AERMOD, were used to assess the concentration of PM_10_, SO_2_, and NO_2_ at seven target locations in case of using HFO and NG scenarios. The results of both models are correlated, in a good conformity, and are factor of 2. Therefore, it is concluded that, in case of uncomplicated (flat) surrounding terrain and low wind speed condition, using Power law scheme to calculate the plume dispersion parameters in CGPM can give accurate results compared to the advanced Gaussian Plume models like AERMOD. The assessment of pollutants’ concentrations indicated that Minya and Qibliya towns are the most vulnerable locations affected by the pollution resulting from Arab Abu Saed Brickworks. The radiation measurement of burning HFO in brick kilns showed that, the average concentrations of Ra-226, Th-232 and K-40 per each kiln reached 7000, 6500, and 8000 Bq/day respectively. However, the average concentrations of naturally occurring radionuclides were considerable high; the average dose due to external exposure to different organs or tissues was 2.65 mSv/y. This value within the international permissible values for workers rather than public and it represents valuable exposure that must be taken into considerations.
